# Unraveling the role of HIF and epigenetic regulation in pulmonary arterial hypertension: implications for clinical research and its therapeutic approach

**DOI:** 10.3389/fmed.2024.1460376

**Published:** 2024-10-10

**Authors:** Ankita Mitra, Dan Yi, Zhiyu Dai, Vinicio de Jesus Perez

**Affiliations:** ^1^Division of Pulmonary and Critical Care, Stanford University, Palo Alto, CA, United States; ^2^Department of Internal Medicine, University of Arizona College of Medicine Phoenix, Phoenix, AZ, United States; ^3^Department of Medicine, Washington University School of Medicine in St. Louis (WashU), St. Louis, MO, United States

**Keywords:** hypoxia inducible factor, epigenetics, pulmonary arterial hypertension, animal model, therapeutics

## Abstract

Pulmonary arterial hypertension (PAH) is characterized by pulmonary vascular remodeling with high pulmonary pressure, which ultimately leads to right heart failure and premature death. Emerging evidence suggests that both hypoxia and epigenetics play a pivotal role in the pathogenesis of PAH development. In this review article, we summarize the current developments in regulation of hypoxia inducible factor (HIF) isoforms in PAH vascular remodeling and the development of suitable animal models for discovery and testing of HIF pathway-targeting PAH therapeutics. In addition, we also discuss the epigenetic regulation of HIF-dependent isoforms in PAH and its therapeutic potential from a new perspective which highlights the importance of HIF isoform-specific targeting as a novel salutary strategy for PAH treatment.

## 1 Introduction

Pulmonary arterial hypertension (PAH) is a rare, life-threatening disorder associated with progressive elevation in pulmonary arterial pressure due to increased pulmonary vasculature resistance that leads to right heart failure and death (1–3). Pulmonary vascular remodeling is the key hallmark of PAH, which is driven by a combination of vasoconstriction, proliferation, inflammation, vascular stiffness, and thrombosis resulting in pulmonary vascular resistance (3). Despite extensive research in this area, the underlying mechanisms of PAH progression are incompletely understood. Current therapies are limited to the targeted pathways that only control vasocontraction, i.e., nitric oxide, prostacyclin and endothelin signaling (4). However, even a combination of therapies only improves symptoms and hemodynamics but fails to alleviate eventual right heart failure. Further understanding of vascular remodeling pathways can facilitate the development of appropriate vascular animal models for drug testing and accelerate the search for novel therapeutics against PAH.

PAH development can be linked to a multitude of pulmonary vascular insults such as hypoxia, genetic factors, environmental factors, drugs and toxins, etc. (4, 5). In addition, several molecular pathways have been reported to be associated with PAH development (5). It is, therefore, essential to identify specific ‘master’ pathways that are key triggers for PAH. Among all, Hypoxia inducible factor (HIF) signaling has been shown to be a crucial pathway in PAH pathogenesis (6, 7). Since the HIF pathway contains an abundance of key signaling molecules, a detailed and systematic analysis of the molecules and regulatory mechanisms involved is vital to facilitate identification of specific therapeutic targets (8). In addition, apart from critical molecular targets it is also necessary to identify cellular phenotypes that contribute to PAH pathogenesis and understand their association with the HIF pathway.

In this review, we will further our understanding of the HIF pathway and its epigenetic regulation while discussing the development of suitable animal models for discovery and testing of HIF pathway-targeting PAH therapeutics.

## 2 HIF in PAH

### 2.1 Biology of HIF signaling; role of hypoxia and inflammation in triggering HIF signaling

**Figure 1:**
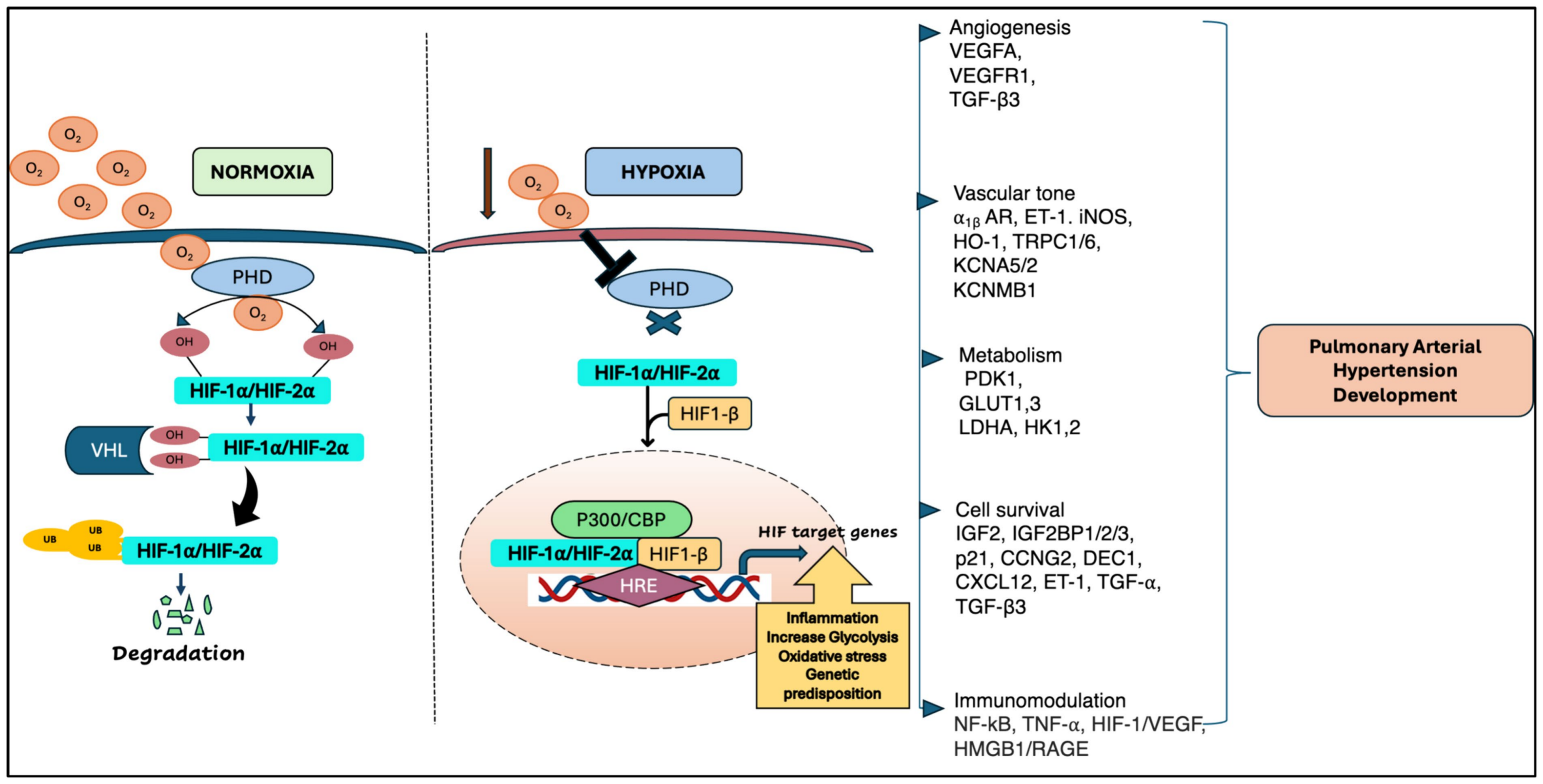
A schematic representation of HIF regulation in pulmonary arterial hypertension [adapted from Wan et al. (133) and Pullamsetti et al. (7)].

HIF is one of the major transcription factors and a master regulator for detecting and adapting to cellular oxygen levels, thereby transcriptionally activating the genes that modulate oxygen homeostasis and metabolic activation. It acts as a heterodimeric complex, composed of the oxygen-sensitive HIF-*α* subunit which includes HIF-1α, HIF-2α (EPAS1) and HIF-3α, and oxygen-insensitive HIF-*β* subunits including HIF-1β [aryl hydrocarbon receptor nuclear translocator (ARNT1, ARNT2, and ARNT3)] (9). Under normoxia, E3 ligase VHL protein binds to the HIF-*α* subunits upon hydroxylation by Prolyl Hydroxylase Domain proteins (PHDs) and Factor Inhibiting HIF (FIH), thus activating the ubiquitin ligase system, and leading to proteasomal degradation of HIF-*α*. However, under hypoxia, the PHDs are inactivated, leading to the attenuation of HIF-a stabilization and dimerization of HIF-1b, which forms an active HIF complex. Upon complex formation, HIF translocate to the nucleus and binds to the E-box-like hypoxia response elements (HREs) to induce gene expression which are involved in various cellular processes such as angiogenesis, erythropoiesis, regulation of vascular tone, cellular metabolism, proliferation, cell survival (9–11) (Figure 1). In pulmonary vasculature, angiogenesis is a repair program where endothelial cells (ECs) form new vessels by angiogenic sprouting which usually occurs after primary vascular plexus formation (9). VEGF is known as a master regulator of angiogenesis and under hypoxia, both VEGF and HIF-1α/HIF-2α are transcriptionally upregulated, promoting angiogenesis (12–14).

Besides oxygen-dependent HIF activity, HIF is also activated by inflammatory cytokines, bacterial products, and growth factors under normoxia conditions (15). To date, among all HIF-*α*, HIF-1α is predominantly expressed in both innate and adaptive immune populations including neutrophils, macrophages, dendritic cells, and lymphocytes (15, 16). In addition, HIF-2α expression is limited to certain range of endothelial cells and tumor associated macrophages including CD8+ T cells under hypoxia conditions (17). The orchestrated processes of HIF regulation modulate inflammation and govern the plethora of signaling pathways and gene expression in numerous physiological responses to hypoxia and the pathogenesis of various lung vascular diseases, including lung cancer, chronic obstructive pulmonary disease (COPD), pulmonary fibrosis, and pulmonary arterial hypertension (PAH).

### 2.2 HIF contribution to PAH: clinical evidence, role in metabolic reprogramming, inflammation, vascular cell senescence, vascular biology, remodeling

Chronic hypoxia is one of the major contributing factors to the development of pathological condition in PAH. It encompasses inflammation, metabolic reprogramming and vascular cell senescence which are governed by gene expression and has an adverse impact on pulmonary vascular remodeling. Several studies support the crucial role of HIFs in chronic hypoxia-induced PAH (7, 18). In PAH, HIFs are involved in the regulation of cell proliferation, migration, and pulmonary vascular remodeling. Activation of HIFs is evident in different categories of PH, including group I PH and PH-associated chronic lung diseases (such as COPD, pulmonary fibrosis, and chronic high-altitude exposure) (19–24). Studies reported that compared to HIF-1α, HIF-2α significantly impacts pulmonary arterial remodeling and the development of PAH (8, 25, 26). Preclinical studies on HIF-2α; inhibitors PT7567 and C76 showed that they reduced the severity of PH in Sugen/hypoxia rats via reducing hemodynamic parameters (8, 27, 28). Recently, overexpression of HIF-2α was reported in lung pericytes of PAH patients (29), highlighting the importance of HIF-2α in pulmonary vasculature remodeling.

Studies show that in PAH, HIF orchestrates the immune/inflammatory dysregulation in response to hypoxia condition which profoundly changes pulmonary arterial endothelial cells (PAECs) and pulmonary arterial smooth muscle cells (PASMCs) phenotype in vascular remodeling (25, 30). The release of inflammatory mediators and inflammatory cells are accelerated by HIF (17). For example, in hypoxia-induced PAH, upon invasion of macrophages and neutrophils, HIF-1α is known to promote macrophage proliferation, chemotaxis and infiltration and induces the release of cytokines (17). In addition, studies reported that HIF and nuclear factor-κB (NF-κB) are the key transcriptional regulator which are jointly involved in the initiation of inflammation of PAH vasculopathy under hypoxia (30, 31). HIF-1*α* expression is upregulated by the p65 and p50 subunits of NF-κB which bind to the HIF-1α promoter (26). In hypoxia-induced PAH, studies show that inhibition of the TNF-α/NF-κB/HIF signaling pathway inhibits angiogenesis via decreasing the HIF-dependent activation (32). Studies also show that increased CD146 expression through NF-κB/HIF mediated cascades promotes synthetic changes in PASMCs which are associated with PAH (27). Taken together, HIF signaling plays crucial roles in regulation of inflammation, metabolic reprogramming, and vascular remodeling in PAH.

Metabolic aberrations have also been identified as a crucial component involved in the disease progression of PAH (28, 29, 33). HIF is the master regulator that controls the metabolic reprogramming in response to hypoxia (33). Studies show that in PAH PAECs, both HIF-1α and HIF-2α contribute to modify the metabolic phenotypes by regulating the expression of mitochondrial enzymes such as pyruvate dehydrogenase kinase 1 (PDK1), lactate dehydrogenase A (LDHA), Hexokinase 1,2 (HK1,2) and Glucose transporter 1,3 (GLUT1,3) to regulate Warburg effect (aerobic glycolysis) and anerobic glycolysis (33, 34). A study reported that increased expression HIF-1α modulates the metabolic shift in the endothelial cells (ECs) of IPAH patients due to decreased nitric oxide (NO) levels with reduced superoxide dismutase activity (SOD) (35). In addition, various studies identified that in PAH, HIF-1α has a close association with mitochondria. It is reported that activation of HIF-1α by cobalt chloride or deferoxamine can lead to mitochondrial fission and subsequent modulation of mitochondrial plasticity in PAH SMCs (36).

In addition, iron deficiency is also one of the metabolic factors reported in PAH population (37). A transcriptional target of HIF, microRNA-210 (miR-210) was found to cause iron deficiency in PAH PAEC via hypoxic repression of iron–sulfur (Fe-S) cluster assembly protein 1 and 2 (ISCU) (33, 38). In contrast, upregulation of miR-210 by HIF-1α increases survival of PASMCs via targeting of the E2F3 transcription factor (39). With regards to HIF-2α, it was observed that patients with Chuvash polycythemia showed HIF-2α gain-of-function mutations associated with PAH development and symptoms such as elevated heart rate and pulmonary ventilation related to metabolic aberrations (40–42). In addition, studies show that octamer-binding transcription factor (OCT4) expression is driven by HIF-2α through miR-130/131-mediated downregulation of peroxisome proliferator-activated receptor-*γ* (PPARγ), resulting in increased proliferation of PAECs and PASMCs in PAH (43).

Another factor which also involved significantly in vascular remodeling in PAH is cellular senescence. Cellular senescence is having a crucial contribution in several vascular diseases, such as coronary artery disease, stroke, myocardial infraction and PAH (44, 45). Mostly the senescent vascular endothelial cells are not identified in the normal lesions; they are predominantly present in the plaque of human atherosclerosis which also leads to the endothelial dysfunction and resulting PAH (46). It has been established that vascular cell senescence plays a major role in the contribution of vascular remodeling and PAH development (47–49). So far, the primarily senescence marker in PAH mainly focuses on p21, p16, p53 and BCl2 and their expression is well studied in animal models as well as in PAH patients (45). However, only few studies reported that HIF signaling also contributes to the vascular senescence in PAH (50). Among HIFs, the crucial role of HIF-1α is primarily observed in endothelial cell (EC) senescence phenotype development and the progression of atherosclerosis (51–54). Emerging evidence reported that HIF-1α and mir-126 plays critical role in EC senescence and it has been proposed that they are new markers of EC senescence progression (55). In addition, other studies revealed that differential expression of HIF-1α/HIF-2α and P53 was identified in PAEC and PASMC in hypoxia-induced PAH animal models, which demonstrated the HIF pathway and P53 crosstalk in the vascular cell senescence and PAH development (50). Hence, based on the above evidence we can demonstrate how HIF pathway is crucial for vascular remodeling and can be used for developing a new drug for the treatment of PAH by targeting HIF pathway and specific HIF animal and cell specific animal models.

### 2.3 HIF-1α vs. HIF-2α: common vs. antagonistic roles in PAH, evidence from animal and cell-specific models

Considering the multitude of cellular and mechanistic roles of HIF contribution in PAH, it is evident that the use of HIF knockout mouse models is a practical approach to gain valuable insights on the HIF pathway involved in hypoxic adaptation of the pulmonary vasculature and PAH development (Table 1). Previously, studies reported that the HIF pathway genes are playing a critical role in embryonic development; however, biallelic deletion in these genes is embryonically lethal (14, 56, 57). For example, double deletion of *Egln1−/−, Hif2a−/−, and Vhl−/−* mice are all embryonically lethal. In contrast, adult mice with heterogenous deletion of *Hif1α and Hif2α* are protected from hypoxia-induced PH development (58, 59). One study from Hu et al. reported that the mice with global deletion of *Hif1α and Hif2α* failed to survive under hypoxia-induced PH (60).

**Table 1:** A summary table for HIF signaling related animal model in PH.

Gene	Genotype	Disease model	Time period of survival	Tissue/cell deletion	RVSP (mm Hg)	RV remodeling	PA remodeling	References
Hif-1α	*Hif1α^−/+^*	Hypoxia	3 weeks	Global/constitutive	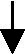	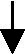	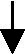	(59)
Hif-1α	*EC Alk^cre^-Hif1α^fl/fl^*	Hypoxia	3 weeks	EC/constitutive	—	—	—	(68)
Hif-1α	*EC Tie2^CreERT^-Hif1-α^fl/fl^*	Hypoxia	3 weeks	EC/inducible (Tam)	—	—	—	(63)
Hif-1α	*SMC SM22α^Cre^-Hif1α^fl/fl^*	Hypoxia	3 weeks	SMC/constitutive	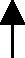	—	NA	(64)
Hif-1α	*EC Cdh5^CreERT^-Hif1α^fl/fl^*	Hypoxia	3 weeks	EC/inducible (Tam)	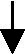	NA	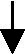	(134)
Hif-1α	*Pdgfrβ^CreERT2^-Hif1α^fl/fl^*	Hypoxia	3 weeks	MC/inducible (Tam)	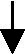	NA	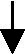	(134)
Hif-1α	*Cx3cr1^Cre^-Hif1α^fl/fl^*	Hypoxia	3 weeks	Mono/constitutive	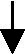	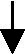	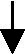	(135)
Hif-1α	*LyzM^Cre^-Hif1α^fl/fl^*	Hypoxia	3 weeks	Myeloid cells	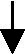	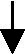	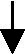	(136)
Hif-1α	*SMC Smm^CreERT2^-Hif1α^fl/fl^*	Hypoxia	4 weeks	SMC/inducible (Tam)	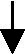	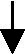	—	(66)
Hif-1α	*Ubc^CreERT^-Hif1α^fl/fl^*	Hypoxia	5 weeks	Global/inducible (Tam)	—	NA	—	(60)
Hif-2α	*EC Alk^Cre^-Hif2α^fl/fl^*	Hypoxia	3 weeks	EC/constitutive	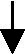	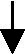	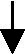	(68)
Hif-2α	*ECTie2^CreERT^-Hif2α^fl/fl^*	Hypoxia	3 weeks	EC/inducible (Tam)	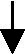	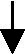	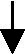	(64)
Hif-2α	*SMC SM^Cre^- Hif2α^fl/fl^*	Hypoxia	3 weeks	SMC/inducible (Tam)	—	NA	—	(63)
Hif-2α	*EC Cdh5^Cre^- Hif2α^fl/fl^*	Hypoxia	4 weeks	EC/constitutive	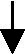	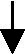	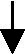	(60, 62)
Hif-2α	*Hif2α ^−/+^*	Hypoxia	4 weeks	Global/constitutive	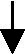	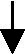	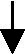	(58)
Hif-2α	*Hif2α ^−/+^*	Hypoxia	4 weeks	Global/constitutive	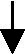	NA	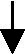	(8, 137)
Hif-2α	*Hif2α ^−/+^*	Hypoxia	4 weeks	Global/constitutive	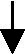	NA	NA	(138)
Hif-2α	Global *Ubc^CreERT^-Hif2α^WT/fl^*	Hypoxia	5 weeks	Global/inducible (Tam)	—	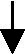	—	(60)
Hif-2α	*EC Cdh5^Cre^-Hif2α^fl/fl^*	Normoxia	3, 6, and 9 months old	EC/constitutive	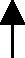	NA	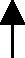	(66)
Hif-2α	*Hif2α^G536w/G536w^*	Normoxia	4–6 months old	Global	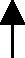	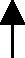	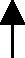	(67)
Hif-2α	*Th^CreERT2^-Hif2α^fl/fl^*	Hypoxia	3 weeks	CAC/constitutive	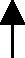	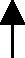	—	(139)
Hif-1α/Hif-2α	*EC Cdh5^Cre-^ Hif1α*/*Hif2α^fl/fl^*	Hypoxia	4 weeks	EC/constitutive	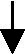	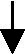	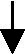	(22)
Hif-1α/Hif-2α	*Hif1α*/*Hif2α^Myh6Cre^*	Hypoxia	4 weeks	Cardiomyocyte/constitutive	—	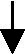	—	(140)
Hif-1α/Hif-2α	*EC Cdh5^Cre-^ Hif1α*/*Hif2α^fl/fl^*	Bleomycin	4 weeks	EC/constitutive	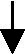	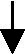	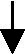	(16)
Egln1	*Egln1^Tie2Cre^*	Normoxia	3 months	EC/constitutive	72	0.85/RV failure	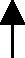	(61)
Egln1	*Egln1^Cdh5Cre^*	Normoxia	3 months	EC/constitutive	50	0.5	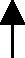	(62)
Egln1	*Egln1^Cdh5Cre^*	Normoxia	15 months	EC/constitutive	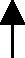	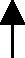	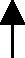	(71)
Egln1/Hif-2α	*Egln1*/*Hif2α^Cdh5Cre^*	Normoxia	3 months	EC/constitutive	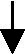	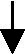	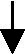	(62)
Egln1/Hif-2α	*Egln1*/*Hif2α^TieCre^*	Normoxia	3 months	EC/constitutive	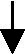	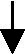	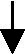	(61)
Vhl	*Vhl^200W^*	Normoxia	7 months	Global	37	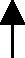	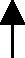	(40)
Vhl	*Vhl^200W^/Hif2α^+/−^*	Normoxia	7 months	Global	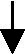	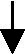	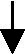	(40)

EC, endothelial cell; SMC, Smooth muscle cell; Tam, Tamoxifen; (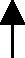) significantly higher compared with control; (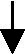) lower than compared with control; (—) no change compared with control; RVSP, right ventricular systolic pressure; PA, pulmonary artery; RV, Right ventricular.

To further dissect the cell-specific role of HIF pathway components in PAH pathogenesis, numerous studies have used cell-specific knockout mouse models to study vascular remodeling. For instance, the PHD2, encoded by EGLN1, is a crucial isoenzyme in normoxia conditions and is a participant in hypoxia related processes such as angiogenesis and cardiac function. Dai et al. and others reported that *Egln1* endothelial conditional knockout mice (*Egln1^EC−/−^*) can spontaneously develop PAH with severe pulmonary vascular remodeling and occlusive pulmonary vascular lesions even in normoxia conditions (61–63), whereas heterogenous *Egln1^EC+/−^* mice shows mild PAH symptoms (63). With regards to *Hif-1α*, a study from Kim et al. shows that mice with constitutive smooth muscle cell (SMC) *Hif-1α* deletion aggravated hypoxia-induced PH (64). Interestingly, another study reported that in mice with SMC-specific *Hif-1α* (inducible) deletion attenuated PH but did not show RV hypertrophy (65).

There is significant progress in the understanding of the role of HIF-2α in the pathogenesis of PAH vascular remodeling (8, 61, 62, 66). Studies report that mice with *HIF2α* G536W (gain-of-functions mutations) develop spontaneous PAH with right ventricle systolic pressure of 66 mm Hg (67). In addition, recent studies reported that endothelial *Hif2α* plays a more significant role in regulating vascular remodeling and PH caused by *Vhl* or *Egln1* deficiency compared to *Hif-1α* (68, 69). The impact of endothelial *Hif-2α* deletion has also been observed on mice with chronic hypoxia-induced PH or *Vhl* or *Egln1* deficiency-induced PH (61, 68, 69). Mice models with Vhl^R200W^ loss of function mutations showed increased susceptibility to PH (40). Similarly, *Egln1^Tie2Cre^* mice progressively develop severe PH at the age of 3.5 months and show 80% mortality by the age of 6 months with increased RV hypertrophy, RV fibrosis, and RV failure (69, 70). In comparison, other studies show that Egln1^Cdh5Cre^ mice display weaker PH pathogenesis (62, 71).

Interestingly, it is observed that the heterozygous deletion of *Hif-2α*, but not *Hif-1α*, in mice with Vhl^R200W^ (loss of function) mutation rescued them from PH development (40). However, other authors show that endothelial deletion of only *Hif-2α* and not *Hif1α* can alleviate PH development in Egln1^Tie2Cre^ or Egln1^Cdh5Cre^ mice (61, 62). Although these studies revealed the association of *HIF-2α* with PH development, the mechanism by which *HIF-2α* exerts its effect is unclear. Moreover, it is also reported that partial deletion of both *HIF-1α* and *HIF-2α* show similar protection from PH development (59). In contrast, Skuti et al. showed that EC Hif2a−/− using Cdh5Cre in mice at 6–9 months ago developed PH (66). One possible reason for this contradictory result May be the upregulation of *HIF-1α* activating factors in smooth muscle by endothelial *HIF-2α* (59). Another contributing factor could be the differences in the roles of *HIF-1α* and *HIF-2α* over a period, evidenced by the reduction of PH rescue in partial *HIF-1α* deletion mice from 3 months to 6 months (61, 62). However, the lack of supporting mechanistic evidence for these observations indicates that there May be additional co-modifiers involved in regulating the activities of *HIF-1α* and *HIF-2α* independently, such as epigenetic factors. However, development of mouse models which incorporate other factors for generating long-lasting and/or irreversible PAH pathology in mice (72). Improved animal models, which well recapitulate irreversible clinical pathology in patients and facilitate development of better therapeutic development and approaches.

## 3 Overview of epigenetic mechanisms and their relevance to PAH

Epigenetic mechanisms play a critical role in PAH, influencing gene expression and phenotype without altering the DNA sequence. Various epigenetic alterations, including DNA methylation, histone modifications, and non-coding RNA dysregulation, contribute to the onset and progression of PAH (73, 74).

One key epigenetic modification observed in PAH is the dysregulation of DNA methylation, which is a process where a methyl group is added to the cytosine residues of DNA, often resulting in gene silencing. In PAH, dysregulation of DNA methylation can lead to the aberrant expression of genes involved in crucial cellular processes. One significant example is the decreased expression of superoxide dismutase 2 (SOD2) due to CpG island methylation. This epigenetic change disrupts redox signaling, leading to the activation of HIF-1α even under normoxic conditions. This inappropriate activation of HIF-1α promotes excessive cell proliferation, which is a hallmark of PAH (75). The chronic proliferation of PASMCs and PAECs contributes to vascular remodeling and the narrowing of the pulmonary arteries, increasing vascular resistance and pressure.

Histone modifications, such as acetylation and methylation, play a significant role in the regulation of chromatin structure and PAH pathogenesis. Histone acetylation and methylation alter the accessibility of the DNA to transcriptional machinery. Changes in histone levels and the expression of histone-modifying enzymes, such as histone deacetylases (HDACs) and bromodomain-containing protein 4 (BRD4), contribute to the abnormal proliferation and resistance to apoptosis in vascular cells. HDACs, for instance, remove acetyl groups from histones, leading to a more condensed chromatin structure and reduced gene expression. In PAH, increased HDAC activity has been linked to the repression of genes that inhibit cell proliferation and promote apoptosis. Abnormal histone modifications can lead to the persistent activation of proliferative pathways and the suppression of apoptotic signals (76). This imbalance contributes to the pathologic remodeling of pulmonary arteries, which is characteristic of PAH progression. Histone modifications can also affect the expression of genes involved in inflammation, fibrosis, and vascular tone, further exacerbating the disease.

Non-coding RNAs, including microRNAs (miRNAs) and long non-coding RNAs (lncRNAs), are crucial regulators of gene expression in PAH. miRNAs are small RNA molecules that can inhibit the translation of target mRNAs or lead to their degradation. Dysregulated miRNAs in PAH can affect pathways related to inflammation, fibrosis, and vascular remodeling. For example, certain miRNAs are known to target and regulate genes involved in SMC proliferation and migration, EC function, and extracellular matrix production. lncRNAs can modulate gene expression at various levels, including chromatin remodeling, transcription, and post-transcriptional processing. In PAH, lncRNAs influence the abnormal proliferation, migration, and survival of pulmonary vascular cells. Some lncRNAs have been found to interact with chromatin-modifying complexes, affecting the expression of genes involved in cell cycle regulation, apoptosis, and inflammation (77). Dysregulation of these lncRNAs contributes to abnormal proliferation, migration, and survival of pulmonary vascular cells. So, targeting epigenetic modifiers holds promise as a therapeutic strategy for PAH.

Understanding the complex interplay of epigenetic mechanisms in PAH provides insights into disease pathogenesis and offers potential avenues for developing novel diagnostic and therapeutic approaches. By elucidating the epigenetic landscape of PAH, it is possible to uncover new treatment strategies that could improve outcomes for patients with this life-threatening condition.

### 3.1 Epigenetic regulation of HIF-dependent genes in PAH

HIF serves as a central regulator in the metabolic reprogramming and mitochondrial dynamics observed in PAH. It exerts control over metabolic enzymes such as pyruvate kinase (PK), pyruvate dehydrogenase kinase (PDK), and pyruvate dehydrogenase (PDH), orchestrating shifts in cellular energy metabolism (78). This metabolic rewiring plays a pivotal role in PAH pathogenesis, influencing cell proliferation and survival. Notably, studies indicate a bidirectional relationship between metabolism and epigenetics in PAH. Epigenetic modifications, including alterations in DNA methylation and histone modifications, can precede changes in metabolism by modulating gene expression, including genes involved in the HIF pathway (79). The stability and transactivity of HIF-*α* are further modulated by its acetylation and methylation. Under hypoxic conditions, histone-modifying enzymes dynamically change the chromatin structure. Some Histone Methyltransferases (HMT) and histone deacetylases (HDACs) induce repressive histone marks. Histone demethylases (HDT), and Histone acetyltransferase (HATs) induce activating marks in chromatin leading to the activation of hypoxia-related genes, including those associated with glycolysis, angiogenesis (80). In the context of PAH, there have been a few studies identified miRNA regulate HIF-1α and HIF-1β, PHD2 leads to dysregulation of HIF signaling (81–83). IRP1, an iron response protein, specifically inhibits HIF-2α via repressing HIF-2α mRNA translation (84). Glycolysis related metabolite and gene, Acetyl-CoA, and PKM2 promote p300, meditate histone acetylation, and promote HIF signaling (85).

In PAH, DNA methylation, particularly hypermethylation, plays a crucial role in vascular pathology. Studies have linked DNA hypermethylation to abnormal cell proliferation and resistance to cell death in the small pulmonary arteries. For instance, Hypermethylation in specific regions of the SOD2 gene was identified, which encodes an enzyme critical for neutralizing harmful superoxide radicals in cells (86). Decreased SOD2 levels are observed in PH, leading to increased oxidative stress and activation of HIF-1α signaling, which promotes abnormal vascular responses such as angiogenesis and inflammation (87). Recent studies have extended this understanding to include epigenetic metabolic changes in right ventricular fibroblasts in PAH. These studies reveal that epigenetic alterations impair mitochondrial function, leading to a pseudo-hypoxic state and activation of HIF-1α, even in normoxic conditions. This dysregulation is associated with abnormal levels of pyruvate dehydrogenase kinase (PDK) isoforms, which promote a metabolic shift known as the Warburg effect. Additionally, upregulation of DNMT1 suppresses SOD2 expression, exacerbating mitochondrial dysfunction and HIF-1α activation. Ultimately, this cascade contributes to increased inflammation and fibrosis in the right ventricle (74).

Hypermethylation of promoters or enhancers of genes encoding key regulators of HIF activity, such as prolyl hydroxylase domain enzymes (PHDs) or von Hippel–Lindau (VHL) tumor suppressor, can impair their expression, leading to sustained HIF activation even under normoxic conditions. Conversely, hypomethylation of HIF-dependent genes May enhance their expression, contributing to pathological processes in PAH. In PAH, pyruvate dehydrogenase kinase 1 (PDK1) inhibits the activity of pyruvate dehydrogenase (PDH), driving a metabolic shift known as the Warburg effect (88). This shift involves a preference for glycolysis over oxidative phosphorylation, leading to increased lactate production even in the presence of oxygen. Additionally, HIF-1α promotes this Warburg shift, further enhancing glycolysis. As a result, there is an increase in the production of inflammatory cytokines like connective tissue growth factor (CTGF) (89) and transforming growth factor beta (TGF-β1) (71, 90) contributing to PAH pathology.

### 3.2 Role of HIF in triggering miRNA and lncRNA

HIF plays a pivotal role in orchestrating the expression of various non-coding RNAs (ncRNAs), including microRNAs (miRNAs) and long non-coding RNAs (lncRNAs), in the context of PAH (91) and other hypoxia-related conditions (92). These ncRNAs, in turn, regulate critical pathways involved in cellular responses to hypoxia, contributing to the progression of PAH and other diseases. One such example is the lncRNA STEAP3-AS1, whose transcription is induced by HIF-1α under hypoxic conditions. STEAP3-AS1, acting as a positive regulator, enhances the expression of STEAP3, thereby promoting colorectal cancer progression (93, 94). This regulatory axis highlights the role of HIF-mediated lncRNA regulation in cancer pathogenesis (93). Furthermore, HIF-1α has been associated with the modulation of exosomal cargo, including miRNAs, which can serve as biomarkers for endothelial senescence progression (95). Dysregulated miRNAs like miR-125a-5p and miR-139-5p have been implicated in EC senescence under hypoxia, suggesting their involvement in PAH pathogenesis (96, 97). Additionally, HIF-1*α* serves as a target for certain miRNAs, such as miR-155, which regulates fibroblast behavior, impacting apoptosis, migration, and proliferation (98). Moreover, intricate networks involving miRNAs, lncRNAs, and core genes like PDGFRB and HIF-1α have been identified, shedding light on their roles in the development of CTEPH (24).

In PAH, dysregulated ncRNAs, including lncRNAs like LINC00963 (99) and 5031425E22Rik/KMT2E-AS1 (100), contribute to disease progression by affecting cellular processes such as proliferation, migration, and metabolic reprogramming. These ncRNAs interact with HIF-mediated pathways, further emphasizing the importance of HIF in regulating ncRNA expression and function in PAH. Overall, the intricate interplay between HIF and ncRNAs underscores their significance in PAH pathogenesis and highlights their potential as therapeutic targets for this debilitating condition.

The dysregulation of HIF signaling is implicated in various pathways relevant to PAH pathology, including vasoconstriction, vascular cell proliferation, metabolic alterations, and inflammation. Among the HIF isoforms, HIF-1α emerges as a key mediator of these effects. It modulates the expression of numerous microRNAs (miRNAs) and, in turn, is reciprocally regulated by miRNAs. For instance, miR-138, whose upregulation is HIF-1α-dependent, is implicated in hypoxia-induced EC dysfunction by regulating nitric oxide (NO) expression via S100A1 (101). Additionally, miR-138 inhibits the hypoxia-induced proliferation of endothelial progenitor cells. These findings underscore the intricate interplay between HIF-1α signaling and miRNA regulation in the context of PAH pathogenesis (78). Moreover, HIF-2α facilitates the expression of nearby genes, specifically the long noncoding RNA (lncRNA) histone lysine N-methyltransferase 2E-antisense 1 (KMT2E-AS1) and histone lysine N-methyltransferase 2E (KMT2E). KMT2E-AS1 helps stabilize the KMT2E protein, leading to an increase in the epigenetic mark histone 3 lysine 4 trimethylation (H3K4me3), which drives HIF-2α-dependent metabolic and pathological endothelial functions. Additionally, this lncRNA pathway boosts HIF-2α expression through epigenetic, transcriptional, and posttranscriptional mechanisms, establishing a positive feedback loop that further enhances HIF-2α activity (102).

### 3.3 Role of HIF in regulating histone modifications and DNA methylation

HIF plays a crucial role in regulating histone modifications and DNA methylation, thereby influencing gene expression under hypoxic conditions. Specifically, HIFs target the expression of proteins associated with modifications of histones, such as histone acetylation, and regulate the balance between DNA methylation and acetylation (103).

In the nucleus, HIFs regulate the expression of numerous genes involved in cellular adaptation to hypoxia, including those encoding proteins associated with histone modifications and DNA methylation (80). HIFs directly target the expression of enzymes involved in histone modifications, such as histone methyltransferases and histone demethylases, as well as enzymes involved in DNA methylation, such as DNA methyltransferases (DNMTs). HIFs influence the balance between histone methylation and acetylation by regulating the expression of histone acetyltransferases (HATs) and histone deacetylases (HDACs). This dynamic regulation of histone modifications by HIFs plays a crucial role in modulating gene expression patterns in response to hypoxia (104, 105).

In the context of ECs, epigenetic modifications, including histone acetylation, contribute to the regulation of placental growth factor (Plgf) expression under hypoxic conditions (106). HIF-1α binds to HREs located in the second intron of Plgf. This binding facilitates the spatial association between the transcriptional start site and the regulatory site within the Plgf gene, potentially enabling efficient gene expression (106). Recent research suggests that transcription factors like HIF-1α can facilitate the formation of chromatin loops, allowing distant regions of DNA to interact effectively (103). This process May occur within specialized nuclear structures known as transcription factories, where genes are organized and looped out from chromosome territories. However, further investigations are needed to fully elucidate the mechanisms by which HIF-1α regulates the three-dimensional chromatin structure of Plgf regulatory regions and how this impacts gene expression in response to hypoxia (7).

Understanding the role of HIF in regulating histone modifications and DNA methylation provides insights into the epigenetic mechanisms underlying cellular responses to hypoxia. By targeting these processes, researchers can develop novel therapeutic strategies for diseases characterized by aberrant hypoxic signaling, such as PAH. Overall, HIFs play a central role in coordinating the cellular response to hypoxia by directly and indirectly regulating histone modifications and DNA methylation. This regulatory network contributes to the adaptation of cells to hypoxic conditions and has implications for various pathological processes, including cancer and PAH (Table 2).

**Table 2:** Known epigenetic downstream targets of HIF-1α, HIF-2α: focus on lncRNA, miRNAs and histone/DNA modifier enzymes discussed in the section.

Epigenetic Target	Description	Role in PAH	Reference(s)
lncRNA MALAT1	Metastasis-Associated Lung Adenocarcinoma Transcript 1	Regulates endothelial cell function and angiogenesis	(141)
lncRNA H19	Imprinted maternally expressed transcript	Promotes pulmonary vascular cell proliferation	(142)
miRNA-210	microRNA-210	miR-210 has an antiapoptotic effect in pulmonary artery smooth muscle cells during hypoxia	(39)
miRNA-17/92 cluster	microRNA cluster including miR-17, miR-18a, miR-19a, miR-20a, miR-19b-1, and miR-92a	Regulates endothelial cell function and angiogenesis	(143, 144)
miRNA-21	microRNA-21	Promotes pulmonary artery smooth muscle cell proliferation and apoptosis	(145)
EZH2	Enhancer of zeste homolog 2	Methyltransferase involved in histone methylation	(102)
HDACs	Histone deacetylases, Epigenetic erasers	Regulate histone acetylation and gene expression	(146)
TET enzymes	Ten-eleven translocation enzymes	DNA demethylases	(147)

These epigenetic targets have been implicated in PAH pathogenesis and are regulated by HIF-1α and/or HIF-2α signaling. They play crucial roles in modulating gene expression, chromatin structure, and cellular processes relevant to PAH, such as proliferation, apoptosis, and angiogenesis.

## 4 Potential therapeutics targeting HIF signaling

### 4.1 Targeting HIF as a therapeutic target in PAH

Targeting HIF signaling holds considerable promise as a therapeutic strategy for PAH and other hypoxia-related conditions. Several potential therapeutics have been proposed, leveraging insights from cancer research (107) and focusing on modulating epigenetic processes (76), metabolomics (108), and antioxidant systems (109) (Table 3). However, controversies surrounding the long-term safety, potential adverse effects, and off-target actions of these drugs highlight the need for further research to elucidate their therapeutic potential and optimize their use in PAH treatment.

**Table 3:** Summary the detail about the HIF targeted drugs, model and treatment protocol.

Drug	Model	Treatment Protocol	Controversies	References
HIF-2α-selective inhibitor, compound 76 (C76)	Egln1^Tie2Cre^ mice, Sugen 5,416/hypoxia-induced PH rats, and monocrotaline-exposed PH rats	Egln1^Tie2Cre^ mice: C76 (compound 76) (12.5 mg/kg body weight, i.p., daily) for 12 weeks; Sugen 5,416/hypoxia rats: C76 (12.5 mg/kg body weight, i.p.) daily for the subsequent 21 days; MCT rats: C76 (12.5 mg/kg, i.p., daily) for 14 days	The controversies surrounding C76, a HIF-2α-selective inhibitor, include its potential differential effects on alveolar development compared to previous HIF inhibition studies, uncertainty regarding its direct action on pulmonary fibroblasts and smooth muscle cells, and questions about its safety despite limited adverse effects observed in animal models.	(8)
Hif-2a-ASO	Hypoxia mice for 5 weeks	i.p. injection twice a week before hypoxia incubation at the dose of 50 mg/kg		(60)
PT2567 (HIF-2α inhibitor) and sildenafil (phosphodiesterase-5 inhibitor)	Sugen5416/hypoxia rat model, MCT rat model	Sugen5416/hypoxia rat model of PH: Prevention Protocol: Rats received either vehicle, PT2567 (100 mg·kg^−1^ or 300 mg·kg^−1^) once daily, or sildenafil (30 mg·kg^−1^) twice daily during hypoxic exposure. Intervention Protocol: Rats were treated with either vehicle, PT2567 (100 mg·kg^−1^) twice daily, or sildenafil (30 mg·kg^−1^) twice daily for 3 weeks after acclimation to normoxia for 24 h. MCT rat model of PH: Animals were allocated to four groups: vehicle nondisease control, vehicle disease control, PT2567 (100 mg·kg^−1^) intervention, and sildenafil (30 mg·kg^−1^) intervention. PT2567 and sildenafil were administered by oral gavage twice daily for 2 weeks post-MCT injection.	The controversies surrounding these treatment protocols may include the specificity and potential off-target effects of HIF-2α inhibition, the translational relevance of findings from animal models to human PAH patients, and the need for further clinical studies to validate the efficacy and safety of PT2567 and sildenafil in human PAH populations.	(148)
MK-6842	Irp1−/− mice, iron deficiency diet; Vhl^R200w^ mice, Irp1−/−/Vhl^R200w^ mice	Oral gavage daily for 5 weeks at 100 mg·kg^−1^		(112)
Topotecan (TPT)	hypoxia-induced PH-associated pulmonary arteriolar remodeling in PH model rats	Rats in the normoxia and the hypoxia group received saline; the rats in the Hypoxia + TPT and Hypoxia + TPT group received TPT by intragastric administration.	Topotecan prevents hypoxia-induced pulmonary arterial hypertension and inhibits hypoxia-inducible factor-1α and TRPC channels	(149)
Prostaglandin E1 (PGE1)	MCT rat model	CM-Dil-labeled MSCs (108/mL × 0.2 mL) were transplanted by injection through the tail vein.	The controversies surrounding the study include the efficacy and safety of combined preconditioning with PGE1 and YC-1 on MSCs, the optimal dosage of these agents for preconditioning, and the long-term outcomes of MSC transplantation in the context of PAH therapy.	(150)
Rosuvastatin	monocrotaline (MCT)-induced PH Rats	Prevention Protocol: Rats received different doses of rosuvastatin (2 and 10 mg/kg/day) for 4 weeks starting from the time of MCT injection. Treatment Protocol: Rats received different doses of rosuvastatin 4 weeks after MCT injection for 4 weeks.	The promising effects of rosuvastatin on monocrotaline-induced pulmonary arterial hypertension in rats raise questions about its translation to humans, optimal dosing, mechanistic understanding, clinical relevance, safety profile, and potential publication bias.	(151)

Multiple preclinical trials have been conducted using PH mouse and rat models (see Table 3). Dai et al. were the first to demonstrate that pharmacological inhibition of HIF-2α, using the translational inhibitor C76, effectively prevents obliterative pulmonary vascular remodeling and right heart failure in three distinct rodent models of severe PH: Egln1Tie2Cre mice, monocrotaline (MCT)-rats, and sugen5416 plus hypoxia (SuHx)-rats. They also observed clear survival benefits in two of these models through HIF-2α inhibition for the first time (110). Hu et al. showed that antisense oligonucleotides targeting Hif2a reduced pulmonary vascular muscularization and right ventricular hypertrophy in hypoxia-exposed mice. The same group also demonstrated that a small molecule HIF2 inhibitor, PT2567, significantly reduced monocyte recruitment, vascular cell proliferation, vessel muscularization, and PH development in hypoxia-exposed rats (60). Macias et al. further supported these findings, showing that global inhibition of HIF-2α reduced pulmonary vascular hemodynamics and remodeling in both Su5416/hypoxia prevention and intervention models, as well as in MCT-exposed rodents (111). Additionally, Ghosh et al. demonstrated that inactivating HIF-2α with the second-generation allosteric inhibitor MK-6482, a FDA approved drug for renal carcinoma, attenuated polycythemia and PH in VhlR200W mice, Irp1-KO mice, and double mutant VhlR200W;Irp1-KO mice with PH. Overall, these studies suggest that inhibiting HIF-2 signaling is a promising therapeutic approach for PAH (112).

#### 4.1.1 Epigenetic modifiers

Similar to anticancer therapies, modifying epigenetic processes in pulmonary vascular cells shows promise for treating PAH (113). This approach involves targeting the epigenetic regulation of genes involved in HIF signaling and other pathways implicated in PAH pathogenesis (74, 114). However, challenges related to targeting specificity and managing off-target effects need to be addressed to maximize the effectiveness of epigenetic modifiers in PAH treatment.

#### 4.1.2 Metabolomics-based interventions

Metabolomics approaches offer opportunities for early detection, personalized dietary interventions, and advanced drug therapies in PAH (115). By targeting metabolic pathways regulated by HIF and other factors under hypoxic conditions, metabolomics-based interventions aim to restore metabolic homeostasis and mitigate disease progression in PAH patients (116).

#### 4.1.3 Antioxidant systems modulation

Antioxidant systems play a crucial role in regulating HIF signaling by modulating levels of reactive oxygen species (ROS) such as hydrogen peroxide (H_2_O_2_) (117, 118). Strategies aimed at modulating antioxidant systems, such as superoxide dismutase 2 (SOD2), can influence HIF-1α activity and downstream signaling pathways. For instance, siSOD2 has been shown to activate HIF-1α, highlighting the potential of targeting antioxidant systems to modulate HIF signaling in PAH (86).

Despite the promising therapeutic prospects, several challenges need to be addressed to translate these approaches into effective treatments for PAH. These include ensuring target specificity, minimizing off-target effects, understanding cell-specific differences in metabolic activity, and optimizing the magnitude of therapeutic interventions to achieve desired outcomes. Collaborative efforts integrating insights from basic research, clinical studies, and computational modeling will be essential for overcoming these challenges and developing effective therapeutics targeting HIF signaling in PAH (119).

### 4.2 Future perspectives on therapeutic targeting of HIF isoforms in PAH

In PAH, dysregulation of HIF signaling is implicated in vascular remodeling and pulmonary vascular dysfunction. Targeting specific HIF isoforms to promote a favorable epigenetic landscape in PAH represents a promising avenue for research and potential treatment development (7). Selective targeting of HIF isoforms could potentially modulate the epigenetic landscape in PAH, leading to beneficial effects such as improved vascular function and inhibition of pathological remodeling. HIF isoforms, particularly HIF-1α and HIF-2α, play critical roles in cellular responses to hypoxia by regulating genes involved in angiogenesis, metabolism, and cell proliferation (7).

Researchers have explored targeting HIF isoforms in cancer therapy, aiming to disrupt tumor growth and metastasis (120, 121). Strategies include small molecule inhibitors, gene silencing techniques, and immunotherapy approaches (122–124). There are several examples from other diseases where HIF inhibitors have shown promise, suggesting potential applicability in treating PAH (125). In certain cancers, aberrant HIF-1α or HIF-2α signaling contributes to tumor progression by promoting angiogenesis and metabolic reprogramming. For example, HIF-1 inhibitors are extensively studied in cancer treatment because of their role in reducing tumor growth, metastasis, and hypoxia-induced drug resistance. For example, PX-478 was tested in a Phase I dose-escalation study involving cancer patients (NCT00522652), demonstrating effective inhibition of HIF-1α and a reasonable safety profile (126). These inhibitors could be useful in PAH by targeting similar pathways of hypoxia and cellular proliferation in pulmonary arteries. In diseases such as idiopathic pulmonary fibrosis (IPF) (110), liver fibrosis (111), and renal fibrosis (127), HIF-1 inhibitors have demonstrated potential in reducing fibrotic tissue formation. Given that PAH involves vascular remodeling and fibrosis, HIF-1 inhibitors could help mitigate these processes. Additionally, HIF-1 is implicated in chronic kidney disease (CKD) progression by promoting inflammation and fibrosis (128, 129). HIF-1 inhibitors have been explored to reduce these effects, suggesting that similar mechanisms might help in PAH, which also involves inflammatory and fibrotic processes. In conditions like myocardial infarction, stroke (130), and ischemic diseases (131, 132), HIF-1 inhibitors have been investigated for their ability to modulate hypoxia responses, potentially reducing tissue damage and improving outcomes. Belzutifan (PT2977), a HIF-2α inhibitor, has been approved for advanced renal cell carcinoma and showed promising efficacy in preclinical PH models. Taken together, HIF inhibitors might be beneficial for patients with PAH.

Given these examples, HIF inhibitors might offer new therapeutic avenues for PAH by targeting hypoxia, inflammation, and fibrosis—key aspects of PAH pathology. However, rigorous preclinical and clinical studies are necessary to confirm their efficacy and safety in PAH. Translating these strategies to PAH would involve understanding the specific roles of HIF isoforms in pulmonary vascular cells and identifying interventions that can selectively target these isoforms without causing adverse effects. Additionally, considering the complex interplay between HIF signaling and other pathways implicated in PAH pathogenesis could provide further insights into potential therapeutic targets and combination therapies. Overall, while there May not yet be direct examples of targeting specific HIF isoforms in PAH, insights from other diseases and ongoing research into HIF signaling hold promise for developing novel therapeutic approaches. These approaches could modulate the epigenetic landscape and improve outcomes in PAH.

## References

[1] Lai YC Potoka KC Champion HC Mora AL Gladwin MT. Pulmonary arterial hypertension: the clinical syndrome. Circ Res. (2014) 115:115–30. doi: 10.1161/CIRCRESAHA.115.301146, PMID: 24951762 PMC4096686

[2] Condon DF Nickel NP Anderson R Mirza S de Jesus Perez VA. The 6th world symposium on pulmonary hypertension: what's old is new. F1000Res. (2019) 8:8. doi: 10.12688/f1000research.18811.1 31249672 PMC6584967

[3] Galie N Palazzini M Manes A. Pulmonary arterial hypertension: from the kingdom of the near-dead to multiple clinical trial meta-analyses. Eur Heart J. (2010) 31:2080–6. doi: 10.1093/eurheartj/ehq152, PMID: 20504865 PMC2930983

[4] Humbert M Lau EM Montani D Jais X Sitbon O Simonneau G. Advances in therapeutic interventions for patients with pulmonary arterial hypertension. Circulation. (2014) 130:2189–208. doi: 10.1161/CIRCULATIONAHA.114.006974 25602947

[5] de Jesus Perez VA. Molecular pathogenesis and current pathology of pulmonary hypertension. Heart Fail Rev. (2016) 21:239–57. doi: 10.1007/s10741-015-9519-2 26694808

[6] Zhu J Zhao L Hu Y Cui G Luo A Bao C. Hypoxia-inducible factor 2-alpha mediated gene sets differentiate pulmonary arterial hypertension. Front Cell Dev Biol. (2021) 9:701247. doi: 10.3389/fcell.2021.701247, PMID: 34422822 PMC8375387

[7] Pullamsetti SS Mamazhakypov A Weissmann N Seeger W Savai R. Hypoxia-inducible factor signaling in pulmonary hypertension. J Clin Invest. (2020) 130:5638–51. doi: 10.1172/JCI137558, PMID: 32881714 PMC7598042

[8] Dai Z Zhu MM Peng Y Machireddy N Evans CE Machado R. Therapeutic targeting of vascular remodeling and right heart failure in pulmonary arterial hypertension with a HIF-2alpha inhibitor. Am J Respir Crit Care Med. (2018) 198:1423–34. doi: 10.1164/rccm.201710-2079OC, PMID: 29924941 PMC6290950

[9] Krock BL Skuli N Simon MC. Hypoxia-induced angiogenesis: good and evil. Genes Cancer. (2011) 2:1117–33. doi: 10.1177/1947601911423654, PMID: 22866203 PMC3411127

[10] Majmundar AJ Wong WJ Simon MC. Hypoxia-inducible factors and the response to hypoxic stress. Mol Cell. (2010) 40:294–309. doi: 10.1016/j.molcel.2010.09.022, PMID: 20965423 PMC3143508

[11] Semenza GL. Hypoxia-inducible factors in physiology and medicine. Cell. (2012) 148:399–408. doi: 10.1016/j.cell.2012.01.021, PMID: 22304911 PMC3437543

[12] Liu Y Cox SR Morita T Kourembanas S. Hypoxia regulates vascular endothelial growth factor gene expression in endothelial cells. Identification of a 5′ enhancer. Circ Res. (1995) 77:638–43. doi: 10.1161/01.RES.77.3.638 7641334

[13] Forsythe JA Jiang BH Iyer NV Agani F Leung SW Koos RD. Activation of vascular endothelial growth factor gene transcription by hypoxia-inducible factor 1. Mol Cell Biol. (1996) 16:4604–13. doi: 10.1128/MCB.16.9.4604, PMID: 8756616 PMC231459

[14] Compernolle V Brusselmans K Acker T Hoet P Tjwa M Beck H. Loss of HIF-2alpha and inhibition of VEGF impair fetal lung maturation, whereas treatment with VEGF prevents fatal respiratory distress in premature mice. Nat Med. (2002) 8:702–10. doi: 10.1038/nm721, PMID: 12053176

[15] McGettrick AF O'Neill LAJ. The role of HIF in immunity and inflammation. Cell Metab. (2020) 32:524–36. doi: 10.1016/j.cmet.2020.08.002 32853548

[16] Bhandari T Nizet V. Hypoxia-inducible factor (HIF) as a pharmacological target for prevention and treatment of infectious diseases. Infect Dis Ther. (2014) 3:159–74. doi: 10.1007/s40121-014-0030-1 25134687 PMC4269623

[17] Palazon A Goldrath AW Nizet V Johnson RS. HIF transcription factors, inflammation, and immunity. Immunity. (2014) 41:518–28. doi: 10.1016/j.immuni.2014.09.008, PMID: 25367569 PMC4346319

[18] Waypa GB Schumacker PT. Roles of HIF1 and HIF2 in pulmonary hypertension: it all depends on the context. Eur Respir J. (2019) 54:1901929. doi: 10.1183/13993003.01929-2019, PMID: 31831673

[19] Wilkins MR Ghofrani HA Weissmann N Aldashev A Zhao L. Pathophysiology and treatment of high-altitude pulmonary vascular disease. Circulation. (2015) 131:582–90. doi: 10.1161/CIRCULATIONAHA.114.006977 25666980

[20] Fu X Zhang F. Role of the HIF-1 signaling pathway in chronic obstructive pulmonary disease. Exp Ther Med. (2018) 16:4553–61. doi: 10.3892/etm.2018.6785, PMID: 30542404 PMC6257248

[21] Aquino-Galvez A Gonzalez-Avila G Jimenez-Sanchez LL Maldonado-Martinez HA Cisneros J Toscano-Marquez F. Dysregulated expression of hypoxia-inducible factors augments myofibroblasts differentiation in idiopathic pulmonary fibrosis. Respir Res. (2019) 20:130. doi: 10.1186/s12931-019-1100-4, PMID: 31234835 PMC6591870

[22] Bryant AJ Carrick RP McConaha ME Jones BR Shay SD Moore CS. Endothelial HIF signaling regulates pulmonary fibrosis-associated pulmonary hypertension. Am J Physiol Lung Cell Mol Physiol. (2016) 310:L249–62. doi: 10.1152/ajplung.00258.2015, PMID: 26637636 PMC4838140

[23] Garcia-Morales LJ Chen NY Weng T Luo F Davies J Philip K. Altered hypoxic-adenosine axis and metabolism in group III pulmonary hypertension. Am J Respir Cell Mol Biol. (2016) 54:574–83. doi: 10.1165/rcmb.2015-0145OC, PMID: 26414702 PMC4821053

[24] Wang M Gu S Liu Y Yang Y Yan J Zhang X. miRNA-PDGFRB/HIF1A-lncRNA CTEPHA1 network plays important roles in the mechanism of chronic thromboembolic pulmonary hypertension. Int Heart J. (2019) 60:924–37. doi: 10.1536/ihj.18-479, PMID: 31204374

[25] Nicolls MR Mizuno S Taraseviciene-Stewart L Farkas L Drake JI Husseini AA. New models of pulmonary hypertension based on VEGF receptor blockade-induced endothelial cell apoptosis. Pulm Circ. (2012) 2:434–42. doi: 10.4103/2045-8932.105031, PMID: 23372927 PMC3555413

[26] Jiang Y Zhu Y Wang X Gong J Hu C Guo B. Temporal regulation of HIF-1 and NF-κB in hypoxic hepatocarcinoma cells. Oncotarget. (2015) 6:9409–19. doi: 10.18632/oncotarget.3352, PMID: 25823824 PMC4496226

[27] Luo Y Teng X Zhang L Chen J Liu Z Chen X. CD146-HIF-1α hypoxic reprogramming drives vascular remodeling and pulmonary arterial hypertension. Nat Commun. (2019) 10:3551. doi: 10.1038/s41467-019-11500-6, PMID: 31391533 PMC6686016

[28] Saygin D Highland KB Farha S Park M Sharp J Roach EC. Metabolic and functional evaluation of the heart and lungs in pulmonary hypertension by gated 2-[18F]-Fluoro-2-deoxy-D-glucose positron emission tomography. Pulm Circ. (2017) 7:428–38. doi: 10.1177/2045893217701917, PMID: 28597761 PMC5467932

[29] Xu W Koeck T Lara AR Neumann D DiFilippo FP Koo M. Alterations of cellular bioenergetics in pulmonary artery endothelial cells. Proc Natl Acad Sci USA. (2007) 104:1342–7. doi: 10.1073/pnas.0605080104, PMID: 17227868 PMC1783136

[30] Ye Y Xu Q Wuren T. Inflammation and immunity in the pathogenesis of hypoxic pulmonary hypertension. Front Immunol. (2023) 14:1162556. doi: 10.3389/fimmu.2023.1162556, PMID: 37215139 PMC10196112

[31] D'Ignazio L Batie M Rocha S. Hypoxia and inflammation in cancer, focus on HIF and NF-κB. Biomedicines. (2017) 5:21. doi: 10.3390/biomedicines5020021 28536364 PMC5489807

[32] Jin F Zheng X Yang Y Yao G Ye L Doeppner TR. Impairment of hypoxia-induced angiogenesis by LDL involves a HIF-centered signaling network linking inflammatory TNFα and angiogenic VEGF. Aging (Albany NY). (2019) 11:328–49. doi: 10.18632/aging.101726, PMID: 30659163 PMC6366960

[33] Chan SY Rubin LJ. Metabolic dysfunction in pulmonary hypertension: from basic science to clinical practice. Eur Respir Rev. (2017) 26:170094. doi: 10.1183/16000617.0094-2017, PMID: 29263174 PMC5842433

[34] Marin-Hernandez A Gallardo-Perez JC Ralph SJ Rodriguez-Enriquez S Moreno-Sanchez R. HIF-1alpha modulates energy metabolism in cancer cells by inducing over-expression of specific glycolytic isoforms. Mini Rev Med Chem. (2009) 9:1084–101. doi: 10.2174/138955709788922610 19689405

[35] Fijalkowska I Xu W Comhair SA Janocha AJ Mavrakis LA Krishnamachary B. Hypoxia inducible-factor1alpha regulates the metabolic shift of pulmonary hypertensive endothelial cells. Am J Pathol. (2010) 176:1130–8. doi: 10.2353/ajpath.2010.090832, PMID: 20110409 PMC2832136

[36] Marsboom G Toth PT Ryan JJ Hong Z Wu X Fang YH. Dynamin-related protein 1-mediated mitochondrial mitotic fission permits hyperproliferation of vascular smooth muscle cells and offers a novel therapeutic target in pulmonary hypertension. Circ Res. (2012) 110:1484–97. doi: 10.1161/CIRCRESAHA.111.263848, PMID: 22511751 PMC3539779

[37] Rhodes CJ Howard LS Busbridge M Ashby D Kondili E Gibbs JSR. Iron deficiency and raised hepcidin in idiopathic pulmonary arterial hypertension: clinical prevalence, outcomes, and mechanistic insights. J Am Coll Cardiol. (2011) 58:300–9. doi: 10.1016/j.jacc.2011.02.057, PMID: 21737024

[38] Chan SY Zhang YY Hemann C Mahoney CE Zweier JL Loscalzo J. MicroRNA-210 controls mitochondrial metabolism during hypoxia by repressing the iron-sulfur cluster assembly proteins ISCU1/2. Cell Metab. (2009) 10:273–84. doi: 10.1016/j.cmet.2009.08.015, PMID: 19808020 PMC2759401

[39] Gou D Ramchandran R Peng X Yao L Kang K Sarkar J. miR-210 has an antiapoptotic effect in pulmonary artery smooth muscle cells during hypoxia. Am J Physiol Lung Cell Mol Physiol. (2012) 303:L682–91. doi: 10.1152/ajplung.00344.2011, PMID: 22886504 PMC3469638

[40] Hickey MM Richardson T Wang T Mosqueira M Arguiri E Yu H. The von Hippel-Lindau Chuvash mutation promotes pulmonary hypertension and fibrosis in mice. J Clin Invest. (2010) 120:827–39. doi: 10.1172/JCI36362, PMID: 20197624 PMC2827942

[41] Culley MK Chan SY. Mitochondrial metabolism in pulmonary hypertension: beyond mountains there are mountains. J Clin Invest. (2018) 128:3704–15. doi: 10.1172/JCI120847, PMID: 30080181 PMC6118596

[42] Formenti F Beer PA Croft QP Dorrington KL Gale DP Lappin TRJ. Cardiopulmonary function in two human disorders of the hypoxia-inducible factor (HIF) pathway: von Hippel-Lindau disease and HIF-2alpha gain-of-function mutation. FASEB J. (2011) 25:2001–11. doi: 10.1096/fj.10-177378, PMID: 21389259 PMC3159892

[43] Bertero T Lu Y Annis S Hale A Bhat B Saggar R. Systems-level regulation of microRNA networks by miR-130/301 promotes pulmonary hypertension. J Clin Invest. (2014) 124:3514–28. doi: 10.1172/JCI74773, PMID: 24960162 PMC4109523

[44] Childs BG Baker DJ Wijshake T Conover CA Campisi J van Deursen JM. Senescent intimal foam cells are deleterious at all stages of atherosclerosis. Science. (2016) 354:472–7. doi: 10.1126/science.aaf6659, PMID: 27789842 PMC5112585

[45] Roger I Milara J Belhadj N Cortijo J. Senescence alterations in pulmonary hypertension. Cells. (2021) 10:12. doi: 10.3390/cells10123456 PMC8700581 34943963

[46] Minamino T Yoshida T Tateno K Miyauchi H Zou Y Toko H. Ras induces vascular smooth muscle cell senescence and inflammation in human atherosclerosis. Circulation. (2003) 108:2264–9. doi: 10.1161/01.CIR.0000093274.82929.22, PMID: 14557365

[47] Tian XL Li Y. Endothelial cell senescence and age-related vascular diseases. J Genet Genomics. (2014) 41:485–95. doi: 10.1016/j.jgg.2014.08.001 25269674

[48] Ramadhiani R Ikeda K Miyagawa K Ryanto GRT Tamada N Suzuki Y. Endothelial cell senescence exacerbates pulmonary hypertension by inducing juxtacrine notch signaling in smooth muscle cells. iScience. (2023) 26:106662. doi: 10.1016/j.isci.2023.106662, PMID: 37192975 PMC10182325

[49] Born E Lipskaia L Breau M Houssaini A Beaulieu D Marcos E. Eliminating senescent cells can promote pulmonary hypertension development and progression. Circulation. (2023) 147:650–66. doi: 10.1161/CIRCULATIONAHA.122.058794, PMID: 36515093

[50] Wang Z Yang K Zheng Q Zhang C Tang H Babicheva A. Divergent changes of p53 in pulmonary arterial endothelial and smooth muscle cells involved in the development of pulmonary hypertension. Am J Physiol Lung Cell Mol Physiol. (2019) 316:L216–l228. doi: 10.1152/ajplung.00538.2017, PMID: 30358436 PMC6383500

[51] Alique M Luna C Carracedo J Ramírez R. LDL biochemical modifications: a link between atherosclerosis and aging. Food Nutr Res. (2015) 59:29240. doi: 10.3402/fnr.v59.29240, PMID: 26637360 PMC4670441

[52] Alique M Ramírez-Carracedo R Bodega G Carracedo J Ramírez R. Senescent microvesicles: a novel advance in molecular mechanisms of atherosclerotic calcification. Int J Mol Sci. (2018) 19:2003. doi: 10.3390/ijms19072003, PMID: 29987251 PMC6073566

[53] Katsuumi G Shimizu I Yoshida Y Minamino T. Vascular senescence in cardiovascular and metabolic diseases. Front Cardiovasc Med. (2018) 5:18. doi: 10.3389/fcvm.2018.00018, PMID: 29556500 PMC5845435

[54] Wang JC Bennett M. Aging and atherosclerosis: mechanisms, functional consequences, and potential therapeutics for cellular senescence. Circ Res. (2012) 111:245–59. doi: 10.1161/CIRCRESAHA.111.261388 22773427

[55] Alique M Bodega G Giannarelli C Carracedo J Ramírez R. MicroRNA-126 regulates hypoxia-inducible factor-1α which inhibited migration, proliferation, and angiogenesis in replicative endothelial senescence. Sci Rep. (2019) 9:7381. doi: 10.1038/s41598-019-43689-3, PMID: 31089163 PMC6517399

[56] Gnarra JR Ward JM Porter FD Wagner JR Devor DE Grinberg A. Defective placental vasculogenesis causes embryonic lethality in VHL-deficient mice. Proc Natl Acad Sci USA. (1997) 94:9102–7. doi: 10.1073/pnas.94.17.9102, PMID: 9256442 PMC23053

[57] Takeda K Ho VC Takeda H Duan LJ Nagy A Fong GH. Placental but not heart defects are associated with elevated hypoxia-inducible factor alpha levels in mice lacking prolyl hydroxylase domain protein 2. Mol Cell Biol. (2006) 26:8336–46. doi: 10.1128/MCB.00425-06, PMID: 16966370 PMC1636770

[58] Brusselmans K Compernolle V Tjwa M Wiesener MS Maxwell PH Collen D. Heterozygous deficiency of hypoxia-inducible factor-2alpha protects mice against pulmonary hypertension and right ventricular dysfunction during prolonged hypoxia. J Clin Invest. (2003) 111:1519–27. doi: 10.1172/JCI15496, PMID: 12750401 PMC155039

[59] Yu AY Shimoda LA Iyer NV Huso DL Sun X McWilliams R. Impaired physiological responses to chronic hypoxia in mice partially deficient for hypoxia-inducible factor 1alpha. J Clin Invest. (1999) 103:691–6. doi: 10.1172/JCI5912, PMID: 10074486 PMC408131

[60] Hu CJ Poth JM Zhang H Flockton A Laux A Kumar S. Suppression of HIF2 signalling attenuates the initiation of hypoxia-induced pulmonary hypertension. Eur Respir J. (2019) 54:1900378. doi: 10.1183/13993003.00378-2019 31515405 PMC6911916

[61] Dai Z Li M Wharton J Zhu MM Zhao YY. Prolyl-4 hydroxylase 2 (PHD2) deficiency in endothelial cells and hematopoietic cells induces Obliterative vascular remodeling and severe pulmonary arterial hypertension in mice and humans through hypoxia-inducible factor-2alpha. Circulation. (2016) 133:2447–58. doi: 10.1161/CIRCULATIONAHA.116.021494, PMID: 27143681 PMC4907810

[62] Kapitsinou PP Rajendran G Astleford L Michael M Schonfeld MP Fields T. The endothelial prolyl-4-hydroxylase domain 2/hypoxia-inducible factor 2 axis regulates pulmonary artery pressure in mice. Mol Cell Biol. (2016) 36:1584–94. doi: 10.1128/MCB.01055-15, PMID: 26976644 PMC4859687

[63] Tang H Babicheva A McDermott KM Gu Y Ayon RJ Song S. Endothelial HIF-2alpha contributes to severe pulmonary hypertension due to endothelial-to-mesenchymal transition. Am J Physiol Lung Cell Mol Physiol. (2018) 314:L256–75. doi: 10.1152/ajplung.00096.2017, PMID: 29074488 PMC5866501

[64] Kim YM Barnes EA Alvira CM Ying L Reddy S Cornfield DN. Hypoxia-inducible factor-1alpha in pulmonary artery smooth muscle cells lowers vascular tone by decreasing myosin light chain phosphorylation. Circ Res. (2013) 112:1230–3. doi: 10.1161/CIRCRESAHA.112.300646, PMID: 23513056 PMC4005857

[65] Ball MK Waypa GB Mungai PT Nielsen JM Czech L Dudley VJ. Regulation of hypoxia-induced pulmonary hypertension by vascular smooth muscle hypoxia-inducible factor-1alpha. Am J Respir Crit Care Med. (2014) 189:314–24. doi: 10.1164/rccm.201302-0302OC, PMID: 24251580 PMC3977726

[66] Skuli N Liu L Runge A Wang T Yuan L Patel S. Endothelial deletion of hypoxia-inducible factor-2alpha (HIF-2alpha) alters vascular function and tumor angiogenesis. Blood. (2009) 114:469–77. doi: 10.1182/blood-2008-12-193581, PMID: 19439736 PMC2714217

[67] Tan Q Kerestes H Percy MJ Pietrofesa R Chen L Khurana TS. Erythrocytosis and pulmonary hypertension in a mouse model of human HIF2A gain of function mutation. J Biol Chem. (2013) 288:17134–44. doi: 10.1074/jbc.M112.444059, PMID: 23640890 PMC3682519

[68] Cowburn AS Crosby A Macias D Branco C Colaço RDDR Southwood M. HIF2alpha-arginase axis is essential for the development of pulmonary hypertension. Proc Natl Acad Sci USA. (2016) 113:8801–6. doi: 10.1073/pnas.1602978113, PMID: 27432976 PMC4978263

[69] Dai Z. Translational potential of hypoxia-inducible factor-2α signaling in pulmonary hypertension In: Zhao Y. editor. Lung biology and pathophysiology. Boca Raton, FL USA: CRC Press (2024). 87–100.

[70] Dai Z Zhao YY. Discovery of a murine model of clinical PAH: mission impossible? Trends Cardiovasc Med. (2017) 27:229–36. doi: 10.1016/j.tcm.2016.12.003, PMID: 28089339 PMC5400690

[71] Wang S Zeng H Xie XJ Tao YK He X Roman RJ. Loss of prolyl hydroxylase domain protein 2 in vascular endothelium increases pericyte coverage and promotes pulmonary arterial remodeling. Oncotarget. (2016) 7:58848–61. doi: 10.18632/oncotarget.11585, PMID: 27613846 PMC5312280

[72] Park CS Kim SH Yang HY Kim JH Schermuly RT Cho YS. <i>Sox17</i> deficiency promotes pulmonary arterial hypertension via HGF/c-met signaling. Circ Res. (2022) 131:792–806. doi: 10.1161/CIRCRESAHA.122.320845, PMID: 36205124 PMC9612711

[73] Ho L Hossen N Nguyen T Vo A Ahsan F. Epigenetic mechanisms as emerging therapeutic targets and microfluidic chips application in pulmonary arterial hypertension. Biomedicines. (2022) 10:170. doi: 10.3390/biomedicines10010170, PMID: 35052850 PMC8773438

[74] Dave J Jagana V Janostiak R Bisserier M. Unraveling the epigenetic landscape of pulmonary arterial hypertension: implications for personalized medicine development. J Transl Med. (2023) 21:477. doi: 10.1186/s12967-023-04339-5, PMID: 37461108 PMC10353122

[75] Kim GH Ryan JJ Marsboom G Archer SL. Epigenetic mechanisms of pulmonary hypertension. Pulm Circ. (2011) 1:347–56. doi: 10.4103/2045-8932.87300, PMID: 22140624 PMC3224426

[76] Chelladurai P Seeger W Pullamsetti SS. Epigenetic mechanisms in pulmonary arterial hypertension: the need for global perspectives. Eur Respir Rev. (2016) 25:135–40. doi: 10.1183/16000617.0036-2016, PMID: 27246590 PMC9487251

[77] Bisserier M Janostiak R Lezoualc'h F Hadri L. Targeting epigenetic mechanisms as an emerging therapeutic strategy in pulmonary hypertension disease. Vasc Biol. (2020) 2:R17–r34. doi: 10.1530/VB-19-0030, PMID: 32161845 PMC7065685

[78] Wu D Dasgupta A Read AD Bentley RET Motamed M Chen KH. Oxygen sensing, mitochondrial biology and experimental therapeutics for pulmonary hypertension and cancer. Free Radic Biol Med. (2021) 170:150–78. doi: 10.1016/j.freeradbiomed.2020.12.452, PMID: 33450375 PMC8217091

[79] Ranasinghe A Schwarz MA. Integrating epigenetics and metabolomics to advance treatments for pulmonary arterial hypertension. Biochem Pharmacol. (2022) 204:115245. doi: 10.1016/j.bcp.2022.115245, PMID: 36096239 PMC12616482

[80] Kim J Lee H Yi SJ Kim K. Gene regulation by histone-modifying enzymes under hypoxic conditions: a focus on histone methylation and acetylation. Exp Mol Med. (2022) 54:878–89. doi: 10.1038/s12276-022-00812-1, PMID: 35869366 PMC9355978

[81] Chen T Zhou Q Tang H Bozkanat M Yuan JXJ Raj JU. miR-17/20 controls prolyl hydroxylase 2 (PHD2)/hypoxia-inducible factor 1 (HIF1) to regulate pulmonary artery smooth muscle cell proliferation. J Am Heart Assoc. (2016) 5:e004510. doi: 10.1161/JAHA.116.004510, PMID: 27919930 PMC5210422

[82] Deng B Du J Hu R. MicroRNA-103/107 is involved in hypoxia-induced proliferation of pulmonary arterial smooth muscle cells by targeting HIF-1β. Life Sci. (2016) 147:117–24. doi: 10.1016/j.lfs.2016.01.043, PMID: 26827991

[83] Yue J Guan J Wang X Zhang L Yang Z Ao Q. MicroRNA-206 is involved in hypoxia-induced pulmonary hypertension through targeting of the HIF-1α/Fhl-1 pathway. Lab Investig. (2013) 93:748–59. doi: 10.1038/labinvest.2013.63, PMID: 23628900

[84] Anderson SA Nizzi CP Chang YI Deck KM Schmidt PJ Galy B. The IRP1-HIF-2α axis coordinates iron and oxygen sensing with erythropoiesis and iron absorption. Cell Metab. (2013) 17:282–90. doi: 10.1016/j.cmet.2013.01.007, PMID: 23395174 PMC3612289

[85] Luo W Hu H Chang R Zhong J Knabel M O'Meally R. Pyruvate kinase M2 is a PHD3-stimulated coactivator for hypoxia-inducible factor 1. Cell. (2011) 145:732–44. doi: 10.1016/j.cell.2011.03.054, PMID: 21620138 PMC3130564

[86] Archer SL Marsboom G Kim GH Zhang HJ Toth PT Svensson EC. Epigenetic attenuation of mitochondrial superoxide dismutase 2 in pulmonary arterial hypertension: a basis for excessive cell proliferation and a new therapeutic target. Circulation. (2010) 121:2661–71. doi: 10.1161/CIRCULATIONAHA.109.916098, PMID: 20529999 PMC2914302

[87] Tian L Wu D Dasgupta A Chen KH Mewburn J Potus F. Epigenetic metabolic reprogramming of right ventricular fibroblasts in pulmonary arterial hypertension: a pyruvate dehydrogenase kinase-dependent shift in mitochondrial metabolism promotes right ventricular fibrosis. Circ Res. (2020) 126:1723–45. doi: 10.1161/CIRCRESAHA.120.316443, PMID: 32216531 PMC7274861

[88] Di R Yang Z Xu P Xu Y. Silencing PDK1 limits hypoxia-induced pulmonary arterial hypertension in mice via the Akt/p70S6K signaling pathway. Exp Ther Med. (2019) 18:699–704. doi: 10.3892/etm.2019.7627, PMID: 31281449 PMC6591493

[89] Higgins DF Biju MP Akai Y Wutz A Johnson RS Haase VH. Hypoxic induction of Ctgf is directly mediated by Hif-1. Am J Physiol Renal Physiol. (2004) 287:F1223–32. doi: 10.1152/ajprenal.00245.2004 15315937

[90] Xia XD Peng YP Lei D Chen WQ. Hypercapnia downregulates hypoxia-induced lysyl oxidase expression in pulmonary artery smooth muscle cells via inhibiting transforming growth factor β(1) signalling. Cell Biochem Funct. (2019) 37:193–202. doi: 10.1002/cbf.3390, PMID: 30917408

[91] Ma C Xu Q Huang S Song J Sun M Zhang J. The HIF-1α/miR-26a-5p/PFKFB3/ULK1/2 axis regulates vascular remodeling in hypoxia-induced pulmonary hypertension by modulation of autophagy. FASEB J. (2023) 37:e22906. doi: 10.1096/fj.202200699RR, PMID: 37052859

[92] Zheng F Chen J Zhang X Wang Z Chen J Lin X. The HIF-1α antisense long non-coding RNA drives a positive feedback loop of HIF-1α mediated transactivation and glycolysis. Nat Commun. (2021) 12:1341. doi: 10.1038/s41467-021-21535-3, PMID: 33637716 PMC7910558

[93] Zhou L Jiang J Huang Z Jin P Peng L Luo M. Hypoxia-induced lncRNA STEAP3-AS1 activates Wnt/β-catenin signaling to promote colorectal cancer progression by preventing m(6)A-mediated degradation of STEAP3 mRNA. Mol Cancer. (2022) 21:168. doi: 10.1186/s12943-022-01638-1, PMID: 35986274 PMC9392287

[94] Na H Li X Zhang X Xu Y Sun Y Cui J. lncRNA STEAP3-AS1 modulates cell cycle progression via affecting CDKN1C expression through STEAP3 in colon cancer. Mol Ther Nucleic Acids. (2020) 21:480–91. doi: 10.1016/j.omtn.2020.06.011, PMID: 32679543 PMC7360886

[95] Shan Y Hou B Wang J Chen A Liu S. Exploring the role of exosomal MicroRNAs as potential biomarkers in preeclampsia. Front Immunol. (2024) 15:1385950. doi: 10.3389/fimmu.2024.1385950, PMID: 38566996 PMC10985148

[96] Zhao H Duan R Wang Q Hu X Zhao Q Wu W. MiR-122-5p as a potential regulator of pulmonary vascular wall cell in idiopathic pulmonary arterial hypertension. Heliyon. (2023) 9:e22922. doi: 10.1016/j.heliyon.2023.e22922, PMID: 38144299 PMC10746431

[97] Wade SM Ohnesorge N McLoughlin H Biniecka M Carter SP Trenkman M. Dysregulated miR-125a promotes angiogenesis through enhanced glycolysis. EBioMedicine. (2019) 47:402–13. doi: 10.1016/j.ebiom.2019.08.043, PMID: 31466915 PMC6796559

[98] Bruning U Cerone L Neufeld Z Fitzpatrick SF Cheong A Scholz CC. MicroRNA-155 promotes resolution of hypoxia-inducible factor 1alpha activity during prolonged hypoxia. Mol Cell Biol. (2011) 31:4087–96. doi: 10.1128/MCB.01276-10, PMID: 21807897 PMC3187364

[99] Yang C Rong R Li Y Cheng M Luo Y. Decrease in LINC00963 attenuates the progression of pulmonary arterial hypertension via microRNA-328-3p/profilin 1 axis. J Clin Lab Anal. (2022) 36:e24383. doi: 10.1002/jcla.24383, PMID: 35349725 PMC9102517

[100] Tai YY Yu Q Tang Y Sun W Kelly NJ Okawa S. Allele-specific control of rodent and human lncRNA KMT2E-AS1 promotes hypoxic endothelial pathology in pulmonary hypertension. Sci Transl Med. (2024) 16:eadd2029. doi: 10.1126/scitranslmed.add2029 38198571 PMC10947529

[101] Pleger ST Harris DM Shan C Vinge LE Chuprun JK Berzins B. Endothelial S100A1 modulates vascular function via nitric oxide. Circ Res. (2008) 102:786–94. doi: 10.1161/CIRCRESAHA.108.172031, PMID: 18292599

[102] Habbout K Omura J Awada C Bourgeois A Grobs Y Krishna V. Implication of EZH2 in the pro-proliferative and apoptosis-resistant phenotype of pulmonary artery smooth muscle cells in PAH: a transcriptomic and proteomic approach. Int J Mol Sci. (2021) 22:2957. doi: 10.3390/ijms22062957, PMID: 33803922 PMC7999120

[103] Yfantis A Mylonis I Chachami G Nikolaidis M Amoutzias GD Paraskeva E. Transcriptional response to hypoxia: the role of HIF-1-associated co-regulators. Cells. (2023) 12:798. doi: 10.3390/cells12050798, PMID: 36899934 PMC10001186

[104] Li G Tian Y Zhu WG. The roles of histone deacetylases and their inhibitors in cancer therapy. Front Cell Dev Biol. (2020) 8:576946. doi: 10.3389/fcell.2020.576946, PMID: 33117804 PMC7552186

[105] Xu Y Zhu Q. Histone modifications represent a key epigenetic feature of epithelial-to-mesenchyme transition in pancreatic cancer. Int J Mol Sci. (2023) 24:4820. doi: 10.3390/ijms24054820 36902253 PMC10003015

[106] Tudisco L Della Ragione F Tarallo V Apicella I D'Esposito M Matarazzo MR. Epigenetic control of hypoxia inducible factor-1α-dependent expression of placental growth factor in hypoxic conditions. Epigenetics. (2014) 9:600–10. doi: 10.4161/epi.27835, PMID: 24504136 PMC4121370

[107] Wigerup C Påhlman S Bexell D. Therapeutic targeting of hypoxia and hypoxia-inducible factors in cancer. Pharmacol Ther. (2016) 164:152–69. doi: 10.1016/j.pharmthera.2016.04.009 27139518

[108] Chumak V Rajacharya GH Singh PK. Metabolomic investigations into hypoxia-mediated metabolic reprogramming of pancreatic cancer cells. Methods Mol Biol. (2024) 2755:191–200. doi: 10.1007/978-1-0716-3633-6_14, PMID: 38319579 PMC10915399

[109] Iacobini C Vitale M Haxhi J Pesce C Pugliese G Menini S. Mutual regulation between redox and hypoxia-inducible factors in cardiovascular and renal complications of diabetes. Antioxidants (Basel). (2022) 11:2183. doi: 10.3390/antiox11112183 36358555 PMC9686572

[110] Brereton CJ Yao L Davies ER Zhou Y Vukmirovic M Bell JA. Pseudohypoxic HIF pathway activation dysregulates collagen structure-function in human lung fibrosis. eLife. (2022) 11:11. doi: 10.7554/eLife.69348 PMC8860444 35188460

[111] Cai J Hu M Chen Z Ling Z. The roles and mechanisms of hypoxia in liver fibrosis. J Transl Med. (2021) 19:186. doi: 10.1186/s12967-021-02854-x, PMID: 33933107 PMC8088569

[112] Ghosh MC Zhang DL Ollivierre WH Noguchi A Springer DA Linehan WM. Therapeutic inhibition of HIF-2α reverses polycythemia and pulmonary hypertension in murine models of human diseases. Blood. (2021) 137:2509–19. doi: 10.1182/blood.2020009138, PMID: 33512384 PMC8109019

[113] Boucherat O Vitry G Trinh I Paulin R Provencher S Bonnet S. The cancer theory of pulmonary arterial hypertension. Pulm Circ. (2017) 7:285–99. doi: 10.1177/2045893217701438, PMID: 28597757 PMC5467931

[114] Chen D Gao W Wang S Ni B Gao Y. Critical effects of epigenetic regulation in pulmonary arterial hypertension. Cell Mol Life Sci. (2017) 74:3789–808. doi: 10.1007/s00018-017-2551-8, PMID: 28573430 PMC11107652

[115] Bassareo PP D'Alto M. Metabolomics in pulmonary hypertension-a useful tool to provide insights into the dark side of a tricky pathology. Int J Mol Sci. (2023) 24:13227. doi: 10.3390/ijms241713227, PMID: 37686034 PMC10487467

[116] Qiu S Cai Y Yao H Lin C Xie Y Tang S. Small molecule metabolites: discovery of biomarkers and therapeutic targets. Signal Transduct Target Ther. (2023) 8:132. doi: 10.1038/s41392-023-01399-3, PMID: 36941259 PMC10026263

[117] Espinosa-Diez C Miguel V Mennerich D Kietzmann T Sánchez-Pérez P Cadenas S. Antioxidant responses and cellular adjustments to oxidative stress. Redox Biol. (2015) 6:183–97. doi: 10.1016/j.redox.2015.07.008, PMID: 26233704 PMC4534574

[118] Yan Q Liu S Sun Y Chen C Yang S Lin M. Targeting oxidative stress as a preventive and therapeutic approach for cardiovascular disease. J Transl Med. (2023) 21:519. doi: 10.1186/s12967-023-04361-7, PMID: 37533007 PMC10394930

[119] Rai N Shihan M Seeger W Schermuly RT Novoyatleva T. Genetic delivery and gene therapy in pulmonary hypertension. Int J Mol Sci. (2021) 22:1179. doi: 10.3390/ijms22031179, PMID: 33503992 PMC7865388

[120] Fallah J Rini BI. HIF inhibitors: status of current clinical development. Curr Oncol Rep. (2019) 21:6. doi: 10.1007/s11912-019-0752-z, PMID: 30671662

[121] Qiu GZ Jin MZ Dai JX Sun W Feng JH Jin WL. Reprogramming of the tumor in the hypoxic niche: the emerging concept and associated therapeutic strategies. Trends Pharmacol Sci. (2017) 38:669–86. doi: 10.1016/j.tips.2017.05.002, PMID: 28602395

[122] Rani S Roy S Singh M Kaithwas G. Regulation of transactivation at C-TAD domain of HIF-1α by factor-inhibiting HIF-1α (FIH-1): a potential target for therapeutic intervention in cancer. Oxidative Med Cell Longev. (2022) 2022:1–21. doi: 10.1155/2022/2407223 PMC9113874 35592530

[123] Lafleur VN Halim S Choudhry H Ratcliffe PJ Mole DR. Multi-level interaction between HIF and AHR transcriptional pathways in kidney carcinoma. Life Sci Alliance. (2023) 6:e202201756. doi: 10.26508/lsa.202201756, PMID: 36725335 PMC9896012

[124] Infantino V Santarsiero A Convertini P Todisco S Iacobazzi V. Cancer cell metabolism in hypoxia: role of HIF-1 as key regulator and therapeutic target. Int J Mol Sci. (2021) 22:5703. doi: 10.3390/ijms22115703, PMID: 34071836 PMC8199012

[125] Camuzi D de Amorim ÍSS Ribeiro Pinto LF Oliveira Trivilin L Mencalha AL Soares Lima SC. Regulation is in the air: the relationship between hypoxia and epigenetics in cancer. Cells. (2019) 8:300. doi: 10.3390/cells8040300, PMID: 30939818 PMC6523720

[126] Tibes R Falchook GS von Hoff DD Weiss GJ Iyengar T Kurzrock R. Results from a phase I, dose-escalation study of PX-478, an orally available inhibitor of HIF-1α. J Clin Oncol. (2010) 28:3076. doi: 10.1200/jco.2010.28.15_suppl.307620479403

[127] Wei X Hou Y Long M Jiang L Du Y. Molecular mechanisms underlying the role of hypoxia-inducible factor-1 α in metabolic reprogramming in renal fibrosis. Front Endocrinol (Lausanne). (2022) 13:927329. doi: 10.3389/fendo.2022.927329, PMID: 35957825 PMC9357883

[128] Locatelli F Minutolo R De Nicola L Del Vecchio L. Evolving strategies in the treatment of anaemia in chronic kidney disease: the HIF-prolyl hydroxylase inhibitors. Drugs. (2022) 82:1565–89. doi: 10.1007/s40265-022-01783-3, PMID: 36350500 PMC9645314

[129] Cochrane Kidney and Transplant Group Natale P Palmer SC Jaure A Hodson EM Ruospo M. Hypoxia-inducible factor stabilisers for the anaemia of chronic kidney disease. Cochrane Database Syst Rev. (2022) 2022:CD013751. doi: 10.1002/14651858.CD013751.pub2, PMID: 36005278 PMC9404697

[130] He Q Ma Y Liu J Zhang D Ren J Zhao R. Biological functions and regulatory mechanisms of hypoxia-inducible factor-1α in ischemic stroke. Front Immunol. (2021) 12:801985. doi: 10.3389/fimmu.2021.801985, PMID: 34966392 PMC8710457

[131] Dong P Li Q Han H. HIF-1α in cerebral ischemia (review). Mol Med Rep. (2022) 25:41. doi: 10.3892/mmr.2021.12557 34878158 PMC8674706

[132] Zhang J Qin Y Martinez M Flores-Bellver M Rodrigues M Dinabandhu A. HIF-1α and HIF-2α redundantly promote retinal neovascularization in patients with ischemic retinal disease. J Clin Invest. (2021) 131:e139202. doi: 10.1172/JCI139202, PMID: 34128478 PMC8203455

[133] Wan JJ Yi J Wang FY Zhang C Dai AG. Expression and regulation of HIF-1a in hypoxic pulmonary hypertension: focus on pathological mechanism and pharmacological treatment. Int J Med Sci. (2024) 21:45–60. doi: 10.7150/ijms.88216, PMID: 38164358 PMC10750340

[134] Sheikh AQ Saddouk FZ Ntokou A Mazurek R Greif DM. Cell autonomous and non-cell autonomous regulation of SMC progenitors in pulmonary hypertension. Cell Rep. (2018) 23:1152–65. doi: 10.1016/j.celrep.2018.03.043, PMID: 29694892 PMC5959296

[135] Yu YA Malakhau Y Yu CA. Nonclassical monocytes sense hypoxia, regulate pulmonary vascular remodeling, and promote pulmonary hypertension. J Immunol. (2020) 204:1474–85. doi: 10.4049/jimmunol.1900239, PMID: 31996456 PMC7065976

[136] Kojima H Tokunou T Takahara Y Sunagawa K Hirooka Y Ichiki T. Hypoxia-inducible factor-1 alpha deletion in myeloid lineage attenuates hypoxia-induced pulmonary hypertension. Physiol Rep. (2019) 7:e14025. doi: 10.14814/phy2.14025, PMID: 30927327 PMC6440913

[137] Peng Y Cui C He Y Ouzhuluobu Zhang H Yang D. Down-regulation of EPAS1 transcription and genetic adaptation of Tibetans to high-altitude hypoxia. Mol Biol Evol. (2017) 34:818–30. doi: 10.1093/molbev/msw280, PMID: 28096303 PMC5400376

[138] Song D Navalsky BE Guan W Ingersoll C Wang T Loro E. Tibetan PHD2, an allele with loss-of-function properties. Proc Natl Acad Sci USA. (2020) 117:12230–8. doi: 10.1073/pnas.1920546117, PMID: 32414920 PMC7275716

[139] Macias D Cowburn AS Torres-Torrelo H Ortega-Saenz P Lopez-Barneo J Johnson RS. HIF-2alpha is essential for carotid body development and function. eLife. (2018) 7:7. doi: 10.7554/eLife.38781 PMC5916566 29671738

[140] Smith KA Waypa GB Dudley VJ Budinger GRS Abdala-Valencia H Bartom E. Role of hypoxia-inducible factors in regulating right ventricular function and remodeling during chronic hypoxia-induced pulmonary hypertension. Am J Respir Cell Mol Biol. (2020) 63:652–64. doi: 10.1165/rcmb.2020-0023OC, PMID: 32692928 PMC7605159

[141] He M Shen J Zhang C Chen Y Wang W Tao K. Long-chain non-coding RNA metastasis-related lung adenocarcinoma transcript 1 (MALAT1) promotes the proliferation and migration of human pulmonary artery smooth muscle cells (hPASMCs) by regulating the MicroRNA-503 (miR-503)/toll-like receptor 4 (TLR4) signal axis. Med Sci Monit. (2020) 26:e923123. doi: 10.12659/MSM.923123 32712618 PMC7377003

[142] Su H Xu X Yan C Shi Y Hu Y Dong L. LncRNA H19 promotes the proliferation of pulmonary artery smooth muscle cells through AT(1)R via sponging let-7b in monocrotaline-induced pulmonary arterial hypertension. Respir Res. (2018) 19:254. doi: 10.1186/s12931-018-0956-z, PMID: 30547791 PMC6295077

[143] Xu J Linneman J Zhong Y Yin H Xia Q Kang K. MicroRNAs in pulmonary hypertension, from pathogenesis to diagnosis and treatment. Biomol Ther. (2022) 12:496. doi: 10.3390/biom12040496, PMID: 35454085 PMC9031307

[144] Caruso P MacLean MR Khanin R McClure J Soon E Southgate M. Dynamic changes in lung microRNA profiles during the development of pulmonary hypertension due to chronic hypoxia and monocrotaline. Arterioscler Thromb Vasc Biol. (2010) 30:716–23. doi: 10.1161/ATVBAHA.109.202028, PMID: 20110569

[145] Sarkar J Gou D Turaka P Viktorova E Ramchandran R Raj JU. MicroRNA-21 plays a role in hypoxia-mediated pulmonary artery smooth muscle cell proliferation and migration. Am J Physiol Lung Cell Mol Physiol. (2010) 299:L861–71. doi: 10.1152/ajplung.00201.2010, PMID: 20693317 PMC3006273

[146] António T Soares-da-Silva P Pires NM Gomes P. Salt-inducible kinases: new players in pulmonary arterial hypertension? Trends Pharmacol Sci. (2022) 43:806–19. doi: 10.1016/j.tips.2022.06.008, PMID: 35851157

[147] D'Addario CA Lanier GM Jacob C Bauer N Hewes JL Bhadra A. Differences in the expression of DNA methyltransferases and demethylases in leukocytes and the severity of pulmonary arterial hypertension between ethnic groups. Physiol Rep. (2022) 10:e15282. doi: 10.14814/phy2.15282, PMID: 35581740 PMC9114656

[148] Macias D Moore S Crosby A Southwood M du X Tan H. Targeting HIF2α-ARNT hetero-dimerisation as a novel therapeutic strategy for pulmonary arterial hypertension. Eur Respir J. (2021) 57:1902061. doi: 10.1183/13993003.02061-2019, PMID: 32972983 PMC7930471

[149] Jiang Y Zhou Y Peng G Liu N Tian H Pan D. Topotecan prevents hypoxia-induced pulmonary arterial hypertension and inhibits hypoxia-inducible factor-1α and TRPC channels. Int J Biochem Cell Biol. (2018) 104:161–70. doi: 10.1016/j.biocel.2018.09.010, PMID: 30266526

[150] Jiang DT Tuo L Bai X Bing WD Qu QX Zhao X. Prostaglandin E1 reduces apoptosis and improves the homing of mesenchymal stem cells in pulmonary arterial hypertension by regulating hypoxia-inducible factor 1 alpha. Stem Cell Res Ther. (2022) 13:316. doi: 10.1186/s13287-022-03011-x, PMID: 35842683 PMC9288720

[151] Li XL Guan RJ Li JJ. Attenuation of monocrotaline-induced pulmonary arterial hypertension in rats by rosuvastatin. J Cardiovasc Pharmacol. (2012) 60:219–26. doi: 10.1097/FJC.0b013e31825cce63, PMID: 22592772

